# Awareness and knowledge of the *Good Samaritan Drug Overdose Act* among people at risk of witnessing an overdose in British Columbia, Canada: a multi-methods cross sectional study

**DOI:** 10.1186/s13011-022-00472-4

**Published:** 2022-05-25

**Authors:** Emma Ackermann, Bradley Kievit, Jessica Xavier, Skye Barbic, Max Ferguson, Alissa Greer, Jackson Loyal, Zahra Mamdani, Heather Palis, Bernie Pauly, Amanda Slaunwhite, Jane A. Buxton

**Affiliations:** 1 School of Population and Public Health, University of British Columbia, 2206 E Mall, Vancouver, BC V6T 1Z8 Canada; 2 British Columbia Centre for Disease Control, 655 W 12th Ave, Vancouver, BC V5Z 4R4 Canada; 3 Occupational Science and Occupational Therapy, University of British Columbia, T325 2211 Wesbrook Mall, Vancouver, BC V6T 2A1 Canada; 4 Providence Health Care Research Institute, 10th Floor 1190 Hornby St, Vancouver, BC V6Z 2K5 Canada; 5 Center for Health Evaluation and Outcome Sciences, St. Paul’s Hospital, 588 1081 Burrard Street, Vancouver, BC V6Z 1Y6 Canada; 6 School of Criminology, Simon Fraser University, 8888 University Drive, Burnaby, BC V5A 1S6 Canada; 7 Centre for Applied Research in Mental Health and Addiction, Simon Fraser University, 2400 515 W Hastings St, Vancouver, BC V6B 5K3 Canada; 8 Department of Psychiatry, University of British Columbia, 2255 Wesbrook Mall, Vancouver, BC V6T 1Z3 Canada; 9 Canadian Institute for Substance Use Research, STN CSC, University of Victoria, Box 1700, Victoria, BC Canada

**Keywords:** Drug overdose, Good samaritan law, Harm reduction, Emergency medical services, Law enforcement, Decriminalization, Implementation, Knowledge

## Abstract

**Introduction:**

Bystanders to drug overdoses often avoid or delay calling 9–1-1 and cite fear of police involvement as a main reason. In 2017, the *Good Samaritan Drug Overdose Act (GSDOA)* was enacted by the Canadian government to provide people present at an overdose with legal protection from charges for simple drug possession, and conditions stemming from simple possession. Few studies have taken a multi-methods approach to evaluating the *GSDOA*. We used quantitative surveys and qualitative interviews to explore awareness, understanding, and perceptions of the *GSDOA* in people at risk of witnessing an overdose.

**Methods:**

Quantitative cross-sectional surveys and qualitative telephone interviews were conducted with adults and youth at risk of witnessing an overdose across British Columbia. Cross-sectional survey participants were recruited at 19 Take Home Naloxone sites and online through Foundry. Multivariable logistic regression models were constructed hierarchically to determine factors associated with *GSDOA* awareness. Telephone interview participants were recruited by research assistants with lived/living experience of substance use. Deductive and inductive thematic analyses were conducted to identify major themes.

**Results:**

Overall, 52.7% (*n* = 296) of the quantitative study sample (*N* = 453) reported being aware of the *GSDOA*. In multivariable analysis, cellphone possession (adjusted odds ratio [AOR] = 2.19; 95% confidence interval [CI] 1.36, 3.54) and having recently witnessed an opioid overdose (AOR = 2.34; 95% CI 1.45, 3.80) were positively associated with *GSDOA* awareness. Young adults (25 – 34 years) were more likely to be aware of the *Act* (AOR = 2.10; 95% CI 1.11, 3.98) compared to youth (16–24 years). Qualitative interviews (*N* = 42) revealed that many overestimated the protections offered by the *GSDOA*. To increase awareness and knowledge of the *Act* among youth, participants recommended adding the *GSDOA* to school curricula and using social media. Word of mouth was suggested to reach adults.

**Conclusion:**

Both awareness and knowledge of the *GSDOA* remain low in BC, with many overestimating the protections the *Act* offers. Dissemination efforts should be led by people with lived/living experience and should target those with limited awareness and understanding of the *Act* as misunderstandings can erode trust in law enforcement and harm reduction policy.

**Supplementary Information:**

The online version contains supplementary material available at 10.1186/s13011-022-00472-4.

## Background

In April 2016, the Province of British Columbia (BC), Canada declared a public health emergency due to an increase in opioid overdose deaths from illicit drug toxicity [1, 2]. Although the implementation and expansion of harm reduction and treatment services across the province, including supervised consumption sites/overdose prevention services, opioid agonist treatment, and take-home naloxone, averted an estimated 3,030 illicit drug toxicity deaths between April 2016 and December 2017, the number of deaths continues to rise [2, 3]. The second public health emergency, declared in March 2020, due to the COVID-19 pandemic worsened the overdose emergency leading to a record high 2,224 illicit drug toxicity deaths in BC in 2021 [2, 4].

BC’s Take Home Naloxone (BCTHN) program has been a core component of the response to the overdose crisis in the province [5]. Naloxone is an opioid antagonist that binds to µ-opioid receptors in the brain with a higher affinity than opioids, thus naloxone can temporarily reverse respiratory depression caused by an opioid overdose [5, 6]. Since the implementation of the BCTHN program in 2012, it has expanded to include 1,945 active distribution locations across the province that offer naloxone kits at no cost and overdose recognition and response training to anyone at risk of witnessing an overdose [7]. Naloxone, however, wears off 30–90 min after administration while opioids may remain in the body longer [8]. Additionally, naloxone is only effective against opioid overdoses and does not reverse the effects of other drugs, such as benzodiazepines. Moreover, in recent years the illicit drug supply has become increasingly toxic, leading to a rise in medical complications associated with overdose events [2]. As such, even in circumstances where naloxone is administered to reverse an opioid overdose, it is recommended to call Emergency Health Services (EHS) due to the risk of the overdose recurring and/or other medical complications [8, 9].

Despite these recommendations, and although most overdoses occur in the presence of bystanders, EHS is not always contacted [10, 11]. In Canada, data collected between 2013 and 2016 demonstrated that bystanders who were trained to use naloxone did not call EHS for 30% to 65% of overdoses; over one-third of respondents cited fear of police involvement as the reason they did not call [12]. Similarly, studies conducted in the United States (US) and Australia found that 52% to 75% of people witnessing an overdose reported concerns about police involvement as a reason not to call [13–15].

To reduce fears around police involvement at overdose events and to encourage bystanders to call EHS, two policies were implemented in BC. In June 2016, the BC EHS introduced a policy to stop routinely notifying the police about overdoses [16]. In May 2017, *the Good Samaritan Drug Overdose Act* (*GSDOA*) was enacted by the Canadian federal government [17]. The *GSDOA* legally protects the caller, the person overdosing, along with bystanders at the scene of an overdose against charges for simple possession (possession of drugs for personal use) [17]. People who are in breach of conditions concerning simple possession, such as those on parole or with conditional sentences, are also protected by the *GSDOA* [17]. However, the *GSDOA* does not offer legal protection against other offences such as outstanding warrants or drug trafficking charges [17]. Although the US does not have a comparable federal law, many States have implemented drug-related Good Samaritan Laws similar to the *GSDOA* [18, 19].

To our knowledge, the majority of studies evaluating the *GSDOA*/drug-related Good Samaritan Laws use quantitative methods [16, 20–30], with, to our knowledge, only two multi- or mixed-methods studies [31, 32]. Different methodologies may influence the findings that are produced. Few qualitative and multi- or mixed-methods studies in this area could limit the depth of findings about peoples’ experiences with and perspectives of the *GSDOA*/drug-related Good Samaritan laws and the nuances of complete understanding of these policies. The majority of existing studies were conducted in the US [20–24, 27–30, 32–37], with six studies assessing the *GSDOA* in the Canadian context [16, 25, 26, 31, 38, 39]. Existing literature predominantly examined *GSDOA*/drug-related Good Samaritan Law awareness, effectiveness, and attitudes rather than understanding of these policies. Conceptually, awareness and knowledge are not interchangeable [40]. While an individual may have heard of the drug-related Good Samaritan Law in their jurisdiction (awareness), they may not have a complete understanding of what legal protections it does and does not provide (knowledge). Knowledge of Good Samaritan Laws is important as past research has found those with a complete understanding had increased odds of calling EHS at overdose events compared to those with an incomplete understanding [24].

When assessing awareness and knowledge of drug-related Good Samaritan Laws, youth have been highlighted as a group of interest. Previous research has shown low awareness and understanding of drug-related Good Samaritan Laws among youth [23, 38]. Exposure and access to harm reduction services and education are also limited among youth in comparison to adults [41, 42]. There are unique challenges youth who use drugs must navigate at overdoses, including concerns around the involvement of parents/guardians and stigma towards youth drug use [38]. In order to provide a comprehensive picture of awareness and knowledge of the *GSDOA* in BC, the current study includes both youth and adults. To address the gaps in the literature, our multi-methods study draws on data from quantitative surveys and qualitative interviews with people aged 16 and older at risk of witnessing an overdose in BC. The aim of this study was to assess awareness, knowledge, and perceptions of the *GSDOA* as well as identify factors associated with awareness. Our findings can be used to inform targeted knowledge translation interventions to promote a complete understanding among different sub-populations of people who use drugs (PWUD) (e.g., PWUD in correctional facilities, youth) who may have limited awareness and/or understanding of the *GSDOA*’s specific tenets (i.e. when it applies and for whom it applies).

## Methods

### Study Design

The current study is part of a multi-component evaluation of the *GSDOA* using qualitative and quantitative approaches. We aimed to address the limitations of a singular methodology by employing more than one method to answer our research question [43, 44]. Mixed- and multi-method approaches differ from one another [43, 45]. In theory and practice, some acrimony exists surrounding what distinguishes the two [46]. While mixed methods research also relies on quantitative and qualitative methodologies and their respective strengths and limitations, mixed methods research promotes the integration of findings at the data analysis stage [47]. Contrarily, multi-methods research does not require integration at this stage [43]. Below we present a multi-methods analysis and interpretation of our findings. Quantitative and qualitative data collection and analyses were completed concurrently but independent of each other. The findings from both approaches were integrated and contrasted at the interpretation stage. Our multi-methods approach strengthens our inferences and findings as we are able to bridge the limitations that exist with either one of these methods in isolation. Study-related ethics approval was obtained through the University of British Columbia Research Ethics Board (# H19-01,842).

### Overall Data Collection

Data for this study were obtained through the BC Centre for Disease Control’s (BCCDC) *GSDOA* Survey and qualitative interviews (See Additional File 1), conducted between October 2020 and April 2021, more than three years after the *GSDOA* was introduced in Canada. The survey, interview guide, consent forms, and recruitment materials were designed with input from a larger evaluation team that included researchers, stakeholders from each BC health region, harm reduction coordinators, people with lived and living experience of substance use (PWLLE), youth organizations, and a youth working group. Foundry, a province-wide network of integrated health and social service centers for young people, was involved at all stages. Members from Foundry’s Youth4Youth advisory group formed a youth working group for our study that contributed at various stages of the *GSDOA* evaluation including study design, survey design and interpretation of findings. Two adult PWLLE advisory groups, Professionals for Ethical Engagement of Peers and Peer2Peer, were also consulted throughout the evaluation [48, 49].

### Study Participants

We recruited people aged 16 years and above who were likely to witness an overdose. Respondents were not limited to PWLLE. For example, participants were eligible if they used substances, were a peer worker, and/or had family or friends that were at risk of overdose as they were likely to witness and respond to an overdose [50–52].

### Quantitative Methods

#### Data Collection

**Fig. 1:**
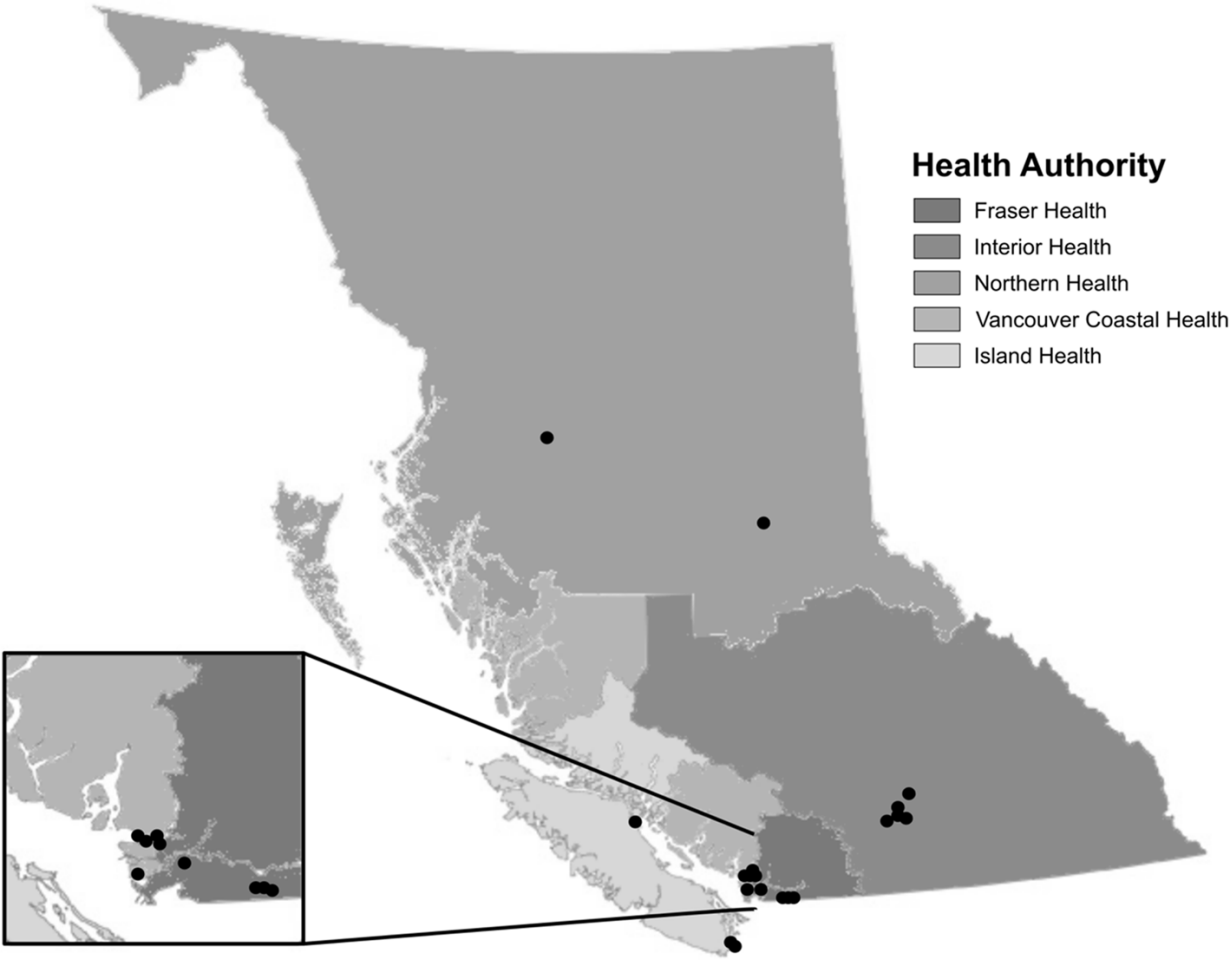
Participating Take Home Naloxone sites of the GSDOA Survey in each of the BC health regions

Regional harm reduction coordinators were first contacted for information on candidate THN sites with sufficient capacity to conduct the study in each health region. Identified sites were contacted and invited to participate in the study, to which 19 THN sites agreed (Fig. 1). Surveys were distributed at participating THN sites across BC to people 16 years and over who picked up a naloxone kit at a THN site. Staff at participating THN sites recruited participants through word of mouth and on-site recruitment posters, provided study information sheets, and administered surveys. Participants were offered $10 CAD honorariums and consent was implied upon filling out the survey. THN sites were offered $5 CAD per enrolled participant in recognition of site resources dedicated to administering surveys (staff time, space). It was important to include youth voices in the survey however, we recognized that youth infrequently access THN sites compared to older age groups For this reason, the survey was also available online through Qualtrics [53], and was advertised by Foundry to youth between 16–24 years old (youth ages defined by United Nations) [54]. With data collection taking place during the COVID-19 pandemic, individuals often came and went quickly from THN sites in order to minimize contact with others. The online survey was therefore also available as an option for those recruited at THN sites who were less willing to interact face-to-face. Online survey participants were entered into a raffle for a 1 in 10 chance of receiving a $50 VISA gift card.

### Study Variables

The primary outcome variable in this study was “*GSDOA* awareness” which was measured by asking participants “*Have you heard about the Good Samaritan Drug Overdose Act?*” to which they could answer one of “yes”, “no”, or “prefer not to say”.

Explanatory variables, outlined in Table 1, included demographic factors and substance use characteristics related to experiencing or witnessing an overdose. Gender, Indigenous self-identification, and perceived risk of experiencing or witnessing were recoded for analysis (See Additional File 1). To assess gender and maintain adequate sample sizes in each level, “trans man”, “trans woman”, and “gender non-binary” were consolidated to “trans and gender expansive”. Indigenous self-identification was similarly consolidated to “Indigenous” and “non-Indigenous” in order to maintain adequate sample sizes. We recognize the diversity of Indigenous peoples and the limitations of using a pan-Indigenous variable but grouped participants in order to communicate meaningful findings. To assess perceived risks, participants were asked to rate the degree to which they felt at risk of experiencing and witnessing an overdose over the past 6 months. Possible responses were “never”, “rarely”, “sometimes”, “often”, or “all the time” and were consolidated to “never” and “ever”.

**Table 1:** Factors associated with *GSDOA* awareness among survey respondents

	**GSDOA Awareness**			*P***-value**^a^
	**Aware **(*N* = 239) *n* (row %)	**Unaware **(*N* = 214) *n* (row %)	**Total **(*N* = 453) *n* (column %)	
**Age (years)**				0.162
16 – 24 years	47 (44.3)	59 (55.7)	106 (23.4)	
25 – 34 years	54 (61.4)	34 (38.6)	88 (19.4)	
35 – 44 years	55 (56.7)	42 (43.3)	97 (21.4)	
45 – 54 years	47 (50.0)	47 (50.0)	94 (20.8)	
55 years and over	30 (51.7)	28 (48.3)	58 (12.8)	
* Unknown*^b^	6 (60.0)	4 (40.0)	10 (2.2)	
**Gender Identity**				0.271
Cis man	135 (52.9)	120 (47.1)	255 (56.3)	
Cis woman	95 (54.6)	79 (45.4)	174 (38.4)	
Trans and gender expansive	8 (36.4)	14 (63.6)	22 (4.9)	
* Unknown*^b^	1 (50.0)	1 (50.0)	2 (0.4)	
**Indigenous Self-Identification**				0.097
Indigenous	81 (47.1)	91 (52.9)	172 (38.0)	
Non-Indigenous	133 (55.9)	105 (44.1)	238 (52.5)	
* Unknown*^b^	25 (58.1)	18 (41.9)	43 (9.5)	
**Health Region**				0.392
Fraser	49 (51.6)	46 (48.4)	95 (21.0)	
Interior	72 (59.0)	50 (41.0)	122 (26.9)	
Island	43 (46.2)	50 (53.8)	93 (20.5)	
Northern	19 (47.5)	21 (52.5)	40 (8.8)	
Vancouver Coastal	56 (54.4)	47 (45.6)	103 (22.7)	
**Housing Status**				0.852
Private	89 (52.7)	80 (47.3)	169 (37.3)	
Supportive or Unstable Housing	112 (54.9)	92 (45.1)	204 (45.0)	
Homeless	35 (51.5)	33 (48.5)	68 (15.0)	
* Unknown*^b^	3 (25.0)	9 (75.0)	12 (2.6)	
**Employment**				0.999
Yes	77 (52.7)	69 (47.3)	146 (32.2)	
No	153 (52.8)	137 (47.2)	290 (64.0)	
* Unknown*^b^	9 (52.9)	8 (47.1)	17 (3.8)	
**Cellphone Possession**				**0.023**
Yes	167 (56.4)	129 (43.6)	296 (65.3)	
No	60 (44.1)	76 (55.9)	136 (30.0)	
* Unknown*^b^	12 (57.1)	9 (42.9)	21 (4.6)	
**Perceived Risk of Experiencing an Overdose** (last 6 months)^c^				** < 0.01**
Never	95 (45.0)	116 (55.0)	211 (46.6)	
Ever	138 (59.7)	93 (40.3)	231 (51.0)	
* Unknown*^b^	6 (54.5)	5 (45.5)	11 (2.4)	
**Perceived Risk of Witnessing an Overdose** (last 6 months)^c^				** < 0.01**
Never	11 (22.0)	39 (78.0)	50 (11.0)	
Ever	222 (56.8)	169 (43.2)	391 (86.3)	
* Unknown*^b^	6 (50.0)	6 (50.0)	12 (2.6)	
**Opioid Use** (last 6 months)				** < 0.01**
Yes	157 (57.7)	115 (42.3)	272 (60.0)	
No	66 (42.6)	89 (57.4)	155 (34.2)	
* Unknown*^b^	16 (61.5)	10 (38.5)	26 (5.7)	
**Opioid Overdose** (last 6 months)^d^				0.224
Yes	53 (63.9)	30 (36.1)	83 (18.3)	
No	99 (55.0)	81 (45.0)	180 (39.7)	
Didn’t use opioids	66 (42.6)	89 (57.4)	155 (34.2)	
* Unknown*^b^	21 (60.0)	14 (40.0)	35 (7.7)	
**Stimulant Overdose** (last 6 months)				0.286
Yes	43 (59.7)	29 (40.3)	72 (15.9)	
No	182 (52.0)	168 (48.0)	350 (77.3)	
* Unknown*^b^	14 (45.2)	17 (54.8)	31 (6.8)	
**Opioid Overdose Witnessed** (last 6 months)				** < 0.01**
Yes	158 (61.5)	99 (38.5)	257 (56.7)	
No	58 (39.7)	88 (60.3)	146 (32.2)	
* Unknown*^b^	23 (46.0)	27 (54.0)	50 (11.0)	
**Stimulant Overdose Witnessed** (last 6 months)				**0.026**
Yes	103 (59.5)	70 (40.5)	173 (38.2)	
No	111 (47.8)	121 (52.2)	232 (51.2)	
* Unknown*^b^	25 (52.1)	23 (47.9)	48 (10.6)	

^a^ Chi square tests exclude participants with unknown explanatory variables

^b^*Unknown* includes missing and “prefer not to say” responses

^c^ “Never” = “Never”; “Ever” = “Rarely/sometimes/often/all the time”

^d^ “Didn’t use opioids” is shown but is not included in the chi square test

### Data Analysis

All analyses were conducted using R version 4.0.2 [55]. Explanatory variables were cross-tabulated with *GSDOA* awareness and chi-square tests were conducted to describe characteristics of the study sample and to explore associations between *GSDOA* awareness and the explanatory variables.

For multivariable analysis, variables were organized into relevant categories, or blocks, that were established by linking concepts through a concept map (See Supplementary Fig. 1, Additional File 2). Hierarchical logistic regression was then used to estimate the association of the variables within each block with *GSDOA* awareness [56]. Hierarchical modelling was used to determine if blocks explained a statistically significant amount of variance in *GSDOA* awareness. The final model was entered block by block in three steps:

1. Demographic characteristics (age, gender, Indigenous self-identification, health region)
2. Overdose response resources (cellphone possession)
3. Overdose characteristics (perceived risk of overdose, perceived risk of witnessing an overdose, stimulant overdose experienced, stimulant overdose witnessed, opioid overdose experienced, opioid overdose witnessed)

In accordance with purposeful model building, explanatory variables where at least one level had a *p*-value < 0.25 in bivariate logistic regression were assessed as candidates for the final model [57, 58]. Variables were then selected through a backwards selection approach based on the lowest Akaike’s information criteria (AIC) value [59]. Variables with conceptual relevance that were excluded by the selection approach (i.e. age and gender) were retained in the model. “Perceived risk of witnessing an overdose” was removed as it was assumed to be a mediator of the association between witnessing an overdose and *GSDOA* awareness. Model fit at each stage was estimated using likelihood ratio R^2^ [60]. Models were compared using the Likelihood Ratio Test with each model being compared to the previous one [57].

A secondary analysis of *GSDOA* knowledge was conducted by exploring the individual survey questions that assessed understanding of *when* and *to whom* the *GSDOA* provides protection.

### Missing data

Covariates included in each analysis were chosen based on their statistical and conceptual relevance. Complete case analysis was used to develop the analytic sample of the multivariable model, which excluded individuals with missing, “prefer not to say” or “I don’t know” responses, which were combined and labelled “unknown”. This resulted in 153 (31.0%) out of the 493 total responses being removed from the analysis for a final total sample of 340 observations. This level of missing data was considered sufficient to require sensitivity analysis by multiple imputation [61]. Observations were assumed to be missing at random and results were verified by running a parallel analysis using ten imputed datasets generated by multiple imputation by chained equation (MICE) using ten cycles each [62].

### Qualitative Methods

#### Data Collection

Participants were recruited by research assistants with lived or living experience of substance use (referred to as Peer Research Assistants (PRAs)). The PRAs used their networks to recruit people aged 16 years or older who were at risk of witnessing an overdose. Targeted youth recruitment was conducted in collaboration with two organizations that provide social programs and health services for youth across BC. Both organizations shared information about the study online and at their physical locations to recruit youth.

The Research Coordinator provided interested participants with a copy of the consent form and answered questions to ensure participants had a full understanding of the study before giving verbal informed consent. Tri-Council Policy Statement: Ethical Conduct for Research Involving Humans (TCPS 2) consent procedures were followed throughout the process [63].

The interviews took place over the phone due to COVID-19 physical distancing guidance. Before each interview began, the interviewer verbally administered a demographic and drug use questionnaire to collect information about factors such as age, gender, Indigeneity, and types of drugs used. A semi-structured interview guide was employed to assess participants’ awareness and knowledge of the *GSDOA*. The interview guide was developed collaboratively, with input from multiple researchers, PWLLE, and youth. Participants were first asked if they had heard about the *Act*, and how they had heard about it. Next, participants were asked to describe what they knew about the *GSDOA*. Regardless of the participants’ answers, the interviewer provided a definition of the *Act* and described when and to whom it applies. Finally, participants were asked to share their perspectives on the effectiveness of the *GSDOA* and give recommendations to increase awareness and knowledge of the *Act*.

Interviews lasted approximately one hour and participants were given a $20 CAD honorarium for their participation. PRAs were paid $25 CAD per hour for recruiting participants and distributing honorariums. The interviews were audio-recorded and transcribed verbatim by a professional transcription service. Transcripts were imported to the qualitative data management software NVivo 12 for coding and analysis [64].

### Data Analysis

A thematic analysis was conducted, guided by Braun and Clark [65] and Nowell et al.’s [66] frameworks. First, three adult and two youth transcripts were independently open-coded by four members of the qualitative analysis team (AG, BP, JB, JX), including the research coordinator who facilitated interviews (JX). They met to compare codes, discuss initial observations, and build a preliminary coding framework. Six team members (AG, BP, JB, SB, ZM, JX) applied the coding framework to two adult and one youth transcript to identify potential gaps or redundancies in the coding framework. Based on the analysis teams’ discussions, revisions were made to the coding framework before four team members (JX, JL, ZM, EA) coded the interviews. Throughout the coding process, for every three transcripts coded independently, one transcript was coded by all members of the coding team to ensure consistency and reliability, and to adjust the framework as needed. For example, the analysis team included youth-specific codes to capture concepts unique to the youth interviews.

The qualitative analysis referenced in the current paper was primarily deductive, as specific questions had been identified during study design (e.g. What do people at risk of witnessing an overdose know about the tenets of the *GSODA*?), and descriptive codes associated with these questions were included in the coding framework. However, an inductive approach was also present as the analysis team remained open to new concepts that might be present in the data. The analysis was mainly descriptive and was expected to complement the quantitative portion during integration at the interpretation stage. The lead author (EA) conducted the analysis by exploring the data with an eye to *GSDOA* awareness, knowledge, and knowledge translation.

After all interviews had been coded, the coding team met and discussed potential themes as well as patterns or recurring concepts. The predetermined codes relevant to each research question were used as a starting point for theme development, with the analysis then expanding to related codes and concepts. Detailed notes were taken throughout the process, summarizing the concepts within codes as well as comparing and contrasting ideas across codes to identify themes and sub-themes. Concept mapping was used to visualize connections and hierarchies among the developing themes. Demographic characteristics (e.g. age, gender, Indigeneity, location) potentially associated with awareness and knowledge were also examined. The analysis process was iterative, involving comparing themes and sub-themes, going back through the codes and raw data, and discussions with the analysis group, all while considering existing literature and the context of BC. The identified themes and sub-themes were discussed with the larger advisory team and Foundry’s Youth4Youth advisory group to receive feedback and ensure the reliability of the findings. The excerpts presented in this paper were chosen in partnership with Youth4Youth to ensure their representativeness.

## Results

### Quantitative Results

#### Participant Characteristics and GSDOA Awareness

A total of 493 persons completed the survey which, after exclusion of missing and “prefer not to say” responses (*n* = 40) for the outcome variable, provided a study sample of 453 for the primary analyses. Of this sample, respondents who reported being aware of the *GSDOA* (*n* = 239, 52.7%) were asked further questions for an analysis of *GSDOA* understanding.

Table 1 shows demographic characteristics stratified according to *GSDOA* awareness with associations evaluated by chi-square tests. Participants were evenly distributed across the geographic health regions of BC apart from a smaller group in the least populous Northern Health region (*n* = 40, 8.8%). Youth (16–24 years) made up the largest age group (*n* = 106, 23.4%) and participants over the age of 55 years made up the smallest age group (*n* = 58, 12.8%). Age was otherwise distributed consistently among the adult groups (Table 1). Over half the participants were cis men (*n* = 255, 56.3%) and 38.0% identified as Indigenous (*n* = 172). Approximately half the respondents were in supportive housing and/or unstably housed (i.e. hotel, motel, rooming house, single room occupancy, shelter) (*n* = 204, 45.0%). Most participants were unemployed (*n* = 290, 64.0%), and/or had a cellphone (*n* = 296, 65.0%).

When asked about overdose characteristics over the past 6 months, approximately half (*n* = 231, 51.0%) of all participants reported feeling at risk (rarely, sometimes, often or all the time vs. never) of experiencing an overdose and 86% (*n* = 391) felt at some risk of witnessing an overdose. In the last 6 months, 60% (*n* = 272) of the respondents reported using opioids, 56.7% (*n* = 257) had witnessed an opioid overdose, and 18% (*n* = 83) reported experiencing an opioid overdose. In the past 6 months, 38% (*n* = 173) of the respondents had witnessed a stimulant overdose whereas 16% (*n* = 72) had experienced a stimulant overdose.

Awareness of the *GSDOA* was significantly higher (*p* < 0.05) among participants with a cellphone compared to those without (56.4% vs. 44.1%), those who felt at risk (rarely, sometimes, often or all the time) of experiencing an overdose compared to those who felt no risk (59.7% vs. 45.0%), those who felt at risk of witnessing an overdose compared to those who felt no risk (56.8% vs. 22.0%), those who reported opioid use compared to those who did not use opioids (57.7% vs. 42.6%), those who witnessed an opioid overdose compared to those who did not witness an opioid overdose (61.5% vs. 39.7%), and those who witnessed an overdose from stimulants compared to those who did not (59.5% vs. 47.8%). Importantly, there was no significant difference in *GSDOA* awareness for those who completed the survey in person compared to online (53.4% vs. 49.4%, *p* = 0.595). Demographic characteristics stratified according to *GSDOA* awareness using imputed data can be found in Supplementary Table 1 (See Additional File 2).

### Factors associated with GSDOA awareness

Unadjusted and adjusted odds ratios for factors associated with *GSDOA* awareness are shown with 95% confidence intervals in Table 2. Conceptually important variables (i.e. age and gender identity) were retained in the model.

**Table 2:** Estimated odds ratios (OR) and adjusted odds ratios (AOR) for predictors of *GSDOA* awareness among participants as determined by hierarchical logistic regression

	**GSDOA Awareness**^**a**^			
	**Simple Bivariate OR (95% CI)**	**Block 1 (Demographics) AOR (95% CI)**	**Block 2 (Overdose Response) AOR (95% CI)**	**Block 3 (Overdose Characteristics) AOR (95% CI)**
**Demographic Characteristics**				
***Age (years)***				
** 16 – 24**	**—**	**—**	**—**	**—**
** 25 – 34**	2.31 (1.24, 4.31) **	2.15 (1.14, 4.07) *	2.77 (1.42, 5.41) **	2.18 (1.09, 4.35) *
** 35 – 44**	1.89 (1.02, 3.51) *	**1.74 (0.92, 3.28)**	2.06 (1.07, 3.96) *	**1.59 (0.81, 3.14)**
** 45 – 54**	**1.35 (0.74, 2.47)**	**1.24 (0.66, 2.32)**	**1.42 (0.75, 2.69)**	**1.21 (0.62, 2.34)**
** 55 + **	**1.43 (0.71, 2.90)**	**1.31 (0.63, 2.71)**	**1.47 (0.70, 3.10)**	**1.36 (0.63, 2.95)**
***Gender***				
** Cis man**	**—**	**—**	**—**	**—**
** Cis woman**	**0.96 (0.63, 1.47)**	**1.02 (0.66, 1.58)**	**0.91 (0.58, 1.42)**	**1.01 (0.63, 1.60)**
** Trans and gender expansive**	**0.37 (0.12, 1.11)**	**0.47 (0.15, 1.47)**	**0.46 (0.15, 1.45)**	**0.54 (0.17, 1.72)**
**Overdose Response Resources**				
***Cellphone possession***				
** Yes**	1.71 (1.10, 2.66) *		2.15 (1.34, 3.47) **	2.36 (1.44, 3.86) ***
** No**	**—**		**—**	**—**
**Overdose Characteristics**				
***Perceived risk of overdose***^**b**^				
** Ever**	1.82 (1.21, 2.75) **			**1.47 (0.93, 2.31)**
** Never**	**—**			**—**
***Opioid overdose witnessed***				
** Yes**	2.62 (1.70, 4.05) ***			2.29 (1.42, 3.70) ***
** No**	**—**			**—**
*** LR Pseudo–R***^***2***^		**0.019**	**0.040**	**0.080**
*** Pseudo–R***^***2***^*** change***		**0.019**	0.021**	0.040 ***

Reference categories are denoted by “—"; *p < 0.05, **p < 0.01, ***p < 0.001

^a^Final model size *N *= 340 after excluding individuals with “unknown” responses for all variables

^b^ “Never” = “Never”; “Ever” = “Rarely/sometimes/often/all the time”

Models were constructed hierarchically to assess the influence of demographic characteristics, resources to respond to overdose events, and overdose characteristics on *GSDOA* awareness. Age, gender, cellphone possession, perceived risk of experiencing an overdose in the past 6 months, and having witnessed an opioid overdose in the past 6 months were retained in the final model. The demographic characteristic block did not significantly explain variance of *GSDOA* awareness relative to a null model (χ^2^ = 10.37, *p* = 0.110). However, results indicated that young adults (25–34 years) had over twice the odds of *GSDOA* awareness compared to youth (16–24 years) (adjusted odds ratio [AOR] = 2.10 [95% confidence interval [CI] 1.11, 3.98]). Including the overdose response resource block (i.e. cellphone possession) significantly improved the fit of the model in explaining variance of *GSDOA* awareness (χ^2^ = 10.16, *p* < 0.01). Participants with a cellphone had over twice the odds of being aware of the *GSDOA* compared to those without (AOR = 2.19 [95% CI 1.36, 3.54]). The addition of the overdose characteristic block further improved the model fit (χ^2^ = 18.91, *p* < 0.01). Those who had witnessed an opioid overdose over the last 6 months had over twice the odds of being aware of the *GSDOA* compared to those who had not (AOR = 2.34 [95% CI 1.45, 3.80]).

The same regression model was made using imputed data (*n *= 493) (See Supplementary Table 2, Additional File 2). A total of *n* = 11 (2.2%) responses were missing data on age, *n* = 4 (0.8%) for gender identity, *n* = 28 (5.7%) for cellphone possession, *n* = 14 (2.8%) for perceived risk of experiencing an overdose, *n* = 62 (12.5%) for having witnessed an opioid overdose, and *n* = 40 (8.1%) for *GSDOA* awareness. The direction and strength of associations from analyses using the imputed data were consistent with those resulting from the complete case analysis.

### Complete understanding of the GSDOA

Participants who reported awareness of the *GSDOA* (*n* = 239) were asked a set of questions to assess complete understanding of the *GSDOA* (Table 3). Only 112 (46.9%) had a complete understanding of *who* is protected and only 77 (32.2%) had a complete understanding of *when* the *GSDOA* provides protection. More specifically, only half of respondents correctly answered that the *GSDOA* does not provide protection for possession of “larger amounts of drugs on them or items (e.g. scale) that may look like they are involved in drug dealing” at an overdose event (50.2%) or for an outstanding warrant for something beyond simple possession (50.6%). Furthermore, less than half of respondents (38.5%) correctly answered that one can be legally arrested for violating a red/no-go zone restriction for a charge beyond simple possession.

**Table 3:** Knowledge of the *GSDOA* among people at risk of witnessing an overdose

	Response^a^		
	**No/Don’t know***n* (%)	**Yes***n* (%)	**Prefer not to say/Missing***n* (%)
**Do you believe the GSDOA protects the following people from being arrested for simple possession of substances (small amount of drugs for own use) at the scene of an overdose?**^**b**^			
(A) The person who calls 9–1-1	80 (33.5)	144 (60.3)	15 (6.3)
(B) The person who overdoses	87 (36.4)	129 (54.0)	23 (9.6)
(C) Anyone at the scene of an overdose	89 (37.2)	132 (55.2)	18 (7.5)
**Imagine there is an overdose in a public space; 9–1-1 is called and the police come to the scene. Do you think the police can legally arrest a person if they:**^**b**^			
(A) Have a larger amount of drugs on them or items (eg. A scale) that may look like they are involved in drug dealing?	105 (43.9)	120 (50.2)	14 (5.9)
(B) Are in a red/no-go zone they received for a previous charge that was not simple drug possession (eg. theft)?	134 (56.1)	92 (38.5)	13 (5.4)
(C) Have an outstanding warrant for something other than simple drug possession (eg. theft)?	107 (44.8)	121 (50.6)	11 (4.6)

^a^Questions were only asked to respondent who reported previous awareness of the *GSDOA* (n = 239)

^b^The correct answer to the outlined questions is “Yes”

### Qualitative Results

#### Participant Characteristics

Semi-structured interviews were completed with 28 adults (aged 25 or older) and 14 youth (aged 16–24 years), for a total of 42 participants. Table 4 displays the characteristics of qualitative participants.

**Table 4:** Characteristics of qualitative interview participants

	**Adults *N* = 28 *n* (%)**	**Youth *N* = 14 *n* (%)**	**Total *N* = 42 *n* (%)**
**Gender**			
Cis Man	10 (35.7%)	4 (28.6%)	14 (33.3%)
Cis Woman	15 (53.6%)	6 (42.9%)	21 (50%)
Trans and Gender Expansive	0 (0%)	3 (21.4%)	3 (7.1%)
* Unknown*	3 (10.7%)	1 (7.1%)	4 (9.5%)
**Age (years)**			
18 or under	0 (0%)	3 (21.4%)	3 (7.1%)
19–24	0 (0%)	10 (71.4%)	10 (23.8%)
25–35	9 (32.1%)	0 (0%)	9 (21.4%)
36–45	8 (28.6%)	0 (0%)	8 (19%)
46–55	5 (17.9%)	0 (0%)	5 (11.9%)
56–65	3 (10.7%)	0 (0%)	3 (7.1%)
* Unknown*	3 (10.7%)	1 (7.1%)	4 (9.5%)
**Indigenous Self-Identification**			
Indigenous^a^	8 (28.6%)	8 (57.1%)	16 (38.1%)
Non-Indigenous	17 (60.7%)	5 (35.7%)	22 (52.4%)
* Unknown*	3 (10.7%)	1 (7.1%)	4 (9.5%)
**Urbanicity**			
Metropolitan	9 (32.1%)	8 (57.1%)	17 (40.5%)
Large Urban	15 (53.6%)	3 (21.4%)	18 (42.9%)
Medium Urban	0 (0%)	1 (7.1%)	1 (2.4%)
Small Urban	4 (14.3%)	0 (0%)	4 (9.5%)
Rural Hub	0 (0%)	1 (7.1%)	1 (2.4%)
* Unknown*	0 (0%)	1 (7.1%)	1 (2.4%)
**Currently use illicit drugs**			
Yes, opioids only	3 (10.7%)	2 (14.3%)	5 (11.9%)
Yes, stimulants only	8 (28.6%)	2 (14.3%)	10 (23.8%)
Yes, opioids and stimulants	10 (35.7%)	3 (21.4%)	13 (31%)
No^b^	4 (14.3%)	6 (42.9%)	10 (23.8%)
* Unknown*	3 (10.7%)	1 (7.1%)	4 (9.5%)
**Peer Worker**			
Yes	11 (39.3%)		11 (26.2%)
No	17 (60.7%)		17 (40.5%)
* Unknown*	0 (0.0%)		0 (0.0%)

^a^Data surrounding First Nation, Métis, and/or Inuit self-identification not available

^b^Participants who did not currently use illicit drugs may have used illicit drugs in the past

Among the adult participants, the largest proportion were cisgender women (53.6%), between the ages of 25–35 (32.1%), currently used illicit drugs (75%), and were from large urban centers (53.6%). Of the adults, 28.6% identified as Indigenous and just over one-third (39.3%) identified as peer workers (PWLLE who use their experience to inform their work). The largest proportion of youth were cisgender women (42.9%), between the ages of 19–24 years (71.4%), identified as Indigenous (57.1%), currently used illicit drugs (50%), and were from metropolitan areas (57.1%).

Below, we present the thematic findings related to *GSDOA* awareness and knowledge. Names of participants have been changed to protect their anonymity. The following three themes were identified: 1) Varied awareness of the *GSDOA*; 2) Varied understanding of the *GSDOA*; and 3) Recommendations to increase awareness and understanding. Table 5 illustrates themes and related sub-themes.

**Table 5:** Identified qualitative themes and sub-themes

Theme	Sub-theme
Awareness of the GSDOA	Inconsistent awareness
	Sources of awareness
Understanding of the GSDOA	General understanding
	Misconceptions about the GSDOA
Recommendations to increase awareness and understanding	School curriculum
	Social media
	Word of mouth and the importance of peers

### Varied awareness of the GSDOA

#### Inconsistent awareness

Awareness of the *Act* among adult participants seemed to vary considerably; while some participants believed that everyone around them was aware, others were confident that awareness was generally limited among people at risk of witnessing an overdose.

*“The majority of people that I’m around they all know about it…the big majority.” (Connor, Adult, Currently uses illicit drugs)*

*“A lot of people probably don’t know about it. I’ve been involved with drugs for years and I’ve never heard of it.” (Lynda, Adult, Currently uses illicit drugs)*

Awareness among youth was similarly varied.

*“I think everybody knows that [GSDOA] now, yeah.” (Sam, Youth, Currently uses illicit drugs)*

*“I don’t think youth know about it. I also, like, I haven’t heard parents talk about it either. I don’t think teachers might know about it because we haven’t had that brought up to us in presentations. So I would say, like, the majority of people, especially, I mean, that are in my circle or, you know, slightly outside, I don’t think they know too much about it.” (Sophia, Youth, Does not currently use illicit drugs)*

Our results suggest that, while current knowledge translation efforts may be reaching some people at risk of witnessing an overdose and their acquaintances, others are not effectively receiving information about the *GSDOA*. We did not observe differences in reported access to information about the *GSDOA* based on participant age, gender, or location.

#### Sources of awareness

Participants became aware of the *GSDOA* through a variety of sources. Many youth and adults became aware of the *Act* through posters at harm reduction sites or shelters, where they worked or accessed services. As Rachel shared: *“I saw it at the OPS [overdose prevention service] posted on the wall, and at the women’s shelter posted on a wall*.” *(Rachel, Youth, Does not currently use illicit drugs)*. Word of mouth was also commonly cited as a source of *GSDOA* information. Others heard about the *Act* through widely available trainings, including naloxone training and high school educational sessions, or through educational sessions offered by substance use treatment programs. However, whether the *GSDOA* was mentioned in trainings and the extent to which it was explained seemed to depend on the instructor and course. Some adult and youth participants remembered that the *Act* had been mentioned, however, the details were not always explained.

*“The recent time of the two-hour naloxone training, they mentioned it there and that’s also something that in our training we bring it up to the high schoolers and everything about the Good Samaritan Act. But they didn’t really go in too much detail.” (Emily, Youth, Does not currently use illicit drugs)*

Other participants attended these trainings but did not recall being taught about the *GSDOA.*

*“The most recent training I received, no, I don’t think I did [learn about the GSDOA].” (Kate, Youth, Does not currently use illicit drugs)*

These divergent experiences showcase the inconsistencies in education, even within structured overdose response courses and trainings.

### Varied understanding of the GSDOA

#### General understanding

Several adult and youth participants demonstrated a general understanding of the *GSDOA:* chiefly, the *Act* provides legal protection at the scene of an overdose. Some also clearly understood the *GSDOA* was implemented to encourage people to call 9–1-1 at the scene of an overdose by reducing concerns about police attendance and arrests. For example, as Megan shared:

*“So my understanding of the Good Samaritan Act is that it’s not supposed to be a punitive approach or not supposed to be any repercussions to a medical emergency like I had stated before. So addressing these as public health issues rather than criminality behind them.” (Megan, Adult, Currently uses illicit drugs)*

For these participants, the *GSDOA* was vaguely associated with legal protection for PWUD – however, the extent and specificities of legal protections afforded by the *GSDOA* were unknown and unarticulated by many. Of those participants who had a general understanding of the *Act*, only a few were able to recall specific protections it afforded. As Sam demonstrated, they were aware of legal protection provided for simple possession, however, they did not mention legal protection for conditions related to simple possession.

*“It’s like people who are present during an emergency response… they’re not able to receive drug charges for possession and things like that if they have to call 9-1-1 for someone who’s overdosed. It’s to prevent people from not calling on very serious situations based on the fear of getting a charge for the drugs that they’re using or the drugs that they have on their person”. (Sam, Youth, Currently uses illicit drugs)*

Even among youth and adults who demonstrated a level of understanding (e.g. understanding that the *GSDOA* provides legal protection for simple possession), nearly every participant was surprised by or made aware of some aspect of the *GSDOA* by the interviewer. Anna was aware that the *GSDOA* provided legal protection for simple possession, but was surprised to learn the *Act* did not protect those with warrants at the scene of an overdose:

*“I never knew that if you had warrants, they would pick up, whatever, whatever other stuff, it was just-- like I never had ever had somebody say that fine line. Oh, it was just to protect from possession.” (Anna, Adult, Does not currently use illicit drugs)*

These discrepancies demonstrate clear misunderstandings or gaps in knowledge, even among youth and adults who were aware of the *GSDOA* and had some level of understanding. While the distinct setting in which the *Act* applies (i.e. at overdose events) was more widely understood, the specific type of legal protection the *GSDOA* grants (i.e. simple possession and conditions related to simple possession) was understood by few participants.

#### Misconceptions about the GSDOA

Among both adult and youth participants who were aware of the *GSDOA*, many misunderstood or had incorrect perceptions of it. Most of all, participants overestimated the legal protections that the *Act* provided, with many believing that it provided complete, blanket protection against arrests at overdose events, including for arrests other than simple possession.

*“You’re given basically a get out of jail free card. Because they’re currently in the state of overdosing and you’re trying to save their life while they’re still alive.” (Daniel, Adult, Currently uses illicit drugs)*

*“So the Good Samaritan Drug Act is you don’t have to worry about when you phone and there’s an overdose. They won’t ever check you for your drugs. They’ll never charge you for anything if you’re trying to save a life and-- yeah. So you won’t be searched. You won’t be charged. You won’t be taken away.” (Jack, Adult, Currently uses illicit drugs)*

These participants expressed a sense of immunity and separation from all legal ramifications (e.g. drug trafficking charges, conditions unrelated to simple possession) at overdose events. They believed that they would be completely protected and have the freedom to leave the situation at any time. Confusion surrounding the specific legal protections covered by the *GSDOA* was common. For example, many adults incorrectly believed that warrants were legally protected under the *Act*.

*“The warrant part was where I was confused because people were, like-- they can’t get you even if you have a warrant. And I’m, like, no, I’m pretty sure if you have a warrant they can.” (Rebecca, Adult, Currently uses illicit drugs)*

As several participants shared, incorrect understanding of the *GSDOA* could cause people to experience confusion should they witness police arresting for offences they believed were legally protected.

*“Then over the time it kind of like-- this novelty kind of wore off because people were still like getting problems because of it. Even though they stopped to help somebody, like I said, they still got rolled, you know.” (Anna, Adult, Does not currently use illicit drugs)*

As Anna described, her trust in the *GSDOA* was reduced due to overestimations of the *Act's* protections, sharing that she felt misguided about the protections the *GSDOA* provides.

*“They thought, well, if I stop to help somebody I’m not going to get in any trouble. But they were still getting into trouble because-- yeah, it’s kind of like almost-- it kind of goes with, like, mistrust ‘cause it’s almost like a lie. It’s almost like a half lie, right, so-- yeah, it wasn’t like a half lie, but they just made it sound like everything was going to be okay.” (Anna, Adult, Does not currently use illicit drugs)*

As these findings indicate, misunderstandings can lead to a cascade of effects including reduced trust in police, the effectiveness of the *GSDOA,* and the effectiveness of related harm reduction informed drug policies (e.g. decriminalization) and the organizational bodies implementing them. Interestingly, participants did not speculate about why misunderstandings about the *GSDOA* were so prevalent.

### Recommendations to increase awareness and understanding

Many adult and youth participants emphasized the importance of increasing awareness and knowledge surrounding the *GSDOA* by making information and training about the *Act* widely available:

*“This is stuff that I would love to take back to my community to say, like, look, this Good Samaritan Drug Overdose Act exists for people that don’t know or people that want to know.” (Mary, Adult, Does not currently use illicit drugs)*

The *Act* was perceived by many as an empowering tool for people at the scene of an overdose and a step in the right direction that warranted being promoted.

Participants were asked for suggestions on how best to increase awareness and understanding surrounding the *GSDOA* among their peers and all those who would benefit from the *Act*. The following three suggestions were repeatedly brought up: school curriculum, social media, and word of mouth.

#### School curriculum

Many youth participants and some adults suggested adding information about the *GSDOA* to secondary school curricula. Participants suggested that information about the *Act*, as well as drug education informed by harm reduction principles, should be taught to all students and youth, and not just youth who are considered “at-risk” of drug use.

*“I believe its grade 10 health education is a graduation requirement. So everyone who’s in the public school system in B.C. has to take that health class. So I think presenting within that health class specifically would help.” (Kate, Youth, Does not currently use illicit drugs)*

Several participants suggested specific course subjects that aligned with education about the *GSDOA*, specifically those that focused on health and those required for graduation to target youth broadly.

A few youth participants acknowledged that parents and guardians may be opposed to adding information about the *GSDOA* and other harm reduction informed subject matter to secondary school curricula due to longstanding perceptions of harm reduction as “enabling” or encouraging drug use.

*“Parents are a barrier for sure. A lot of people think, like, oh, if we just don’t talk about it, like, our child will never be in that situation.” (Emily, Youth, Does not currently use illicit drugs)*

Youth participants described being viewed as immature and naïve by adults around them, with the paternalistic desire to shelter youth from the realities of drug use serving as a barrier to drug education.

*“I think we leave them out a lot of the time ‘cause we have a society that feels teenagers are incompetent. Or we feel that educating them encourages drug use. Which is absolutely not the truth. Kids are going to us drugs either way.” (Kate, Youth, Does not currently use illicit drugs)*

As these quotes illustrate, participants expressed opposition to the ideology of abstinence-only education and advocated for the need to challenge these perceptions to make information about the *GSDOA* widely accessible to young people.

#### Social media

In the interviews, social media was a topic brought up especially by youth relative to adults. In addition to suggesting the addition of *GSDOA* education to school curricula, youth recommended that knowledge translation initiatives harness social media to reach diverse audiences:

*“Social media….would be, like, a big one. ‘Cause a lot-- younger kids and, like, my age and a bit younger, we love social media. We’re always on social media.” (Lisa, Youth, Does not currently use illicit drugs)*

Both adult and youth participants pointed to the high level of engagement many youth have with social media platforms and suggested that utilizing these platforms for public health education, such as education surrounding the *GSDOA*, may be the most direct and effective route for reaching a wide audience of young people.

Participants 25 years old and over seemed to express greater apprehension towards using social media to inform and educate adults about the *GSDOA*. One reason for this apprehension may be a lack of access to necessary technology, such as phones or the Internet, for some people:

*“I mean, there’s a lot of people down here that have telephones. But they might not have Internet access.” (Paul, Adult, Currently uses illicit drugs)*

These participants discussed the barriers to technology that some PWUD face due to low socioeconomic status and/or reduced access to resources. As Paul suggests in the quote above, even in cases where people own a cellphone, economic or other circumstances may prevent them from having certain features on their device, such as internet access.

#### Word of Mouth and the Importance of Peers

Among adults, word of mouth was highly recommended. As Mary remarked:

*“For the people on the street I think word of mouth is probably the best right now. Because not a lot of them have cell phones. Not a lot of them have, like, access to social media.” (Mary, Adult, Does not currently use illicit drugs)*

This highlights the accessibility of word of mouth, especially for PWUD who cannot easily access educational materials online or who do not frequent harm reduction sites where informational posters may be displayed. Many participants reported hearing about the *GSDOA* by word of mouth, reinforcing its importance in knowledge translation.

Both adult and youth participants overwhelmingly expressed the importance of involving PWLLE in all efforts to increase awareness and knowledge of the *GSDOA*. Peer educators' ability to foster trusting learning environments free of judgement or stigmatization was considered vital. Youth participants preferred that *GSDOA* educational sessions be facilitated by other youth or by trusted outreach workers with whom they had existing relationships. Stigmatizing and discriminatory attitudes towards youth who use drugs were highlighted as barriers to harm reduction informed drug education, and, as such, being taught by peers was seen as advantageous for increasing comfort, relatability, and transparency between educators and learners.

*“I think it’s important to be taught by peers because it seems a little less intimidating and when you’re youth you tend to think like, you know, adults are in a whole different world and whatnot.” (Emily, Youth, Does not currently use illicit drugs)*

*“Maybe send like outreach workers to tell them. I don’t know… who they trust, you know, and who wouldn’t actually lie to them, right.” (Andrew, Youth, Does not currently use illicit drugs)*

Similarly, Mary, an Indigenous participant, expressed that she would feel more comfortable learning about the Act from Indigenous peers:

*“Having an Indigenous representative connect with an Indigenous person. Because that’s how I find it’s-- being Indigenous, it’s more trusting to trust your own kind of people.” (Mary, Adult, Does not currently use illicit drugs)*

As demonstrated above, the trust and relatability associated with shared identity and experiences was considered equally as important as the content of knowledge translation materials and trainings, as comfort between learners and educators was expected to increase the reach and effectiveness of educational initiatives around the *GSDOA*.

## Discussion

The present study sought to assess awareness, knowledge, and perceptions of the *GSDOA* as well as identify factors associated with awareness of the *Act*. Our quantitative survey revealed that approximately 50% of participants were aware of the *GSDOA*. Of survey participants who were aware, only half had a complete understanding of *who* is protected by the *GSDOA* and only a third had a complete understanding of *when* the *GSDOA* applies. Both our quantitative and qualitative findings revealed that many participants overestimated the legal protections offered by the *GSDOA*, with some qualitative participants incorrectly believing that it protects people with warrants or provides blanket protection from any arrest. Quantitative results indicated greater odds of awareness among those who had a cellphone, those who witnessed an opioid overdose in the last 6 months, and among young adults (aged 25–34 years) compared to youth (aged 16–24 years). In particular, the addition of cellphone possession and overdose characteristics (perceived risk of overdose, opioid overdose witnessed) accounted for a significant portion of the variation in *GSDOA* awareness. Interview participants also shared recommendations to increase awareness and knowledge of the *Act.* While adding *GSDOA* education to school curricula and sharing information on social media were recommended to raise awareness and knowledge among youth, word of mouth was favoured by adults. In addition, participants emphasized the value of peer-led education and recommended involving PWLLE in all knowledge translation strategies.

The finding that just over half of participants reported being aware of the *GSDOA* is consistent with existing research that demonstrates low awareness among people at risk of experiencing or witnessing an overdose [23, 26, 67]. For instance, US studies conducted with people who use opioids in Washington state and Rhode Island demonstrated that only one third to just under half were aware of their state’s drug-related Good Samaritan Law [23, 67], while a previous survey conducted in BC demonstrated that just over half of participants were aware of the *GSDOA* [26]. Importantly, some have argued that awareness measurements alone do not serve as an adequate indicator and that complete understanding of the tenets of drug-related Good Samaritan Laws should be assessed [37, 40]. Indeed, as Jakubowski et al. [24] found, those who had a complete understanding of the law had over three times the odds of calling EHS at an overdose compared to those who had an incomplete understanding.

Beyond awareness, both our quantitative and qualitative findings demonstrate that many people do not have an accurate understanding of the *GSDOA* and the legal protections it provides. These findings are comparable to results from two US studies and two studies conducted in BC [24, 26, 29, 68]. Notably, a study from BC found that only 45% and 61% of respondents who were aware of the *GSDOA* had a complete understanding of when and to whom protection was offered, respectively [26]. Respondents were classified as having underestimated the *GSDOA* if they incorrectly answered the questions pertaining to *who* is protected, and classified as having overestimated the *GSDOA* if they responded incorrectly to *when* protection is offered. Both our quantitative and qualitative results indicate a greater proportion of respondents overestimated the protections provided under the *GSDOA*. Our interviews shed light on the particular tenets of the *Act* which are overestimated, such as incorrectly believing that people with outstanding warrants were legally protected. Others believed that the *Act* was a universal ‘get out of jail free card’ at overdose events. The overestimations present in our quantitative and qualitative findings are concerning as misunderstandings of the *GSDOA* may place people at risk of arrest for offences they believed were legally protected, which can sow distrust in police and the *Act* itself. Our findings are in line with other studies such as a study conducted in Vancouver, BC that found 32.6% of participants who used drugs overestimated the *GSDOA* and a similar proportion (37.2%) of participants underestimated the *Act*, particularly among those who reported negative past experiences with police officers [68]. A qualitative study conducted with police officers across BC found that many officers were unaware or had incomplete understanding of the *GSDOA* [39]. It was also revealed that officers rely heavily on their discretion to interpret and implement the *GSDOA*, leading to inconsistent applications and, in some cases, punitive policing responses at overdose events. Overestimation of the *GSDOA*’s protections in combination with inconsistent enforcement may reduce the effectiveness of the *GSDOA*. Moreover, long-term misunderstandings of the *Act* may cause continuous erosion of trust in law enforcement, government, and harm reduction policy as a whole; damaging the effectiveness of future policies (e.g. broader decriminalization). Accurate understanding and implementation of the *GSDOA* is therefore vital to encourage people to call EHS.

Awareness and complete understanding of the *GSDOA* varied according to participant characteristics. In our study, youth and adults were initially analyzed separately however, beyond the points discussed here, there were few meaningful differences between the two groups. After careful consideration, youth and adults were combined and analyzed together. Results from our multivariable model indicate that youth had lower odds of being aware of the *GSDOA* compared to adults 25 – 34 years old. Multiple studies have demonstrated low awareness and understanding of local Good Samaritan Laws in youth [23, 38]. Youth awareness and knowledge of the *GSDOA* may differ from that of adults due to the unique barriers youth face. As qualitative participants shared, abstinence-only drug education from parents and other adults as well as stigma around youth drug use can restrict knowledge in this age group. Research demonstrates that harm reduction informed education and services may be less accessible to youth in comparison to adults [41, 42]. Future initiatives, such as *GSDOA* awareness campaigns, should be mindful of this and prioritize youth as a group at risk of witnessing overdoses through knowledge translation activities that are engaging and that leverage settings that youth are connected to (e.g. Foundry centres in BC). More research is needed to determine youths’ access to information about the *GSDOA*, and, more broadly, youths’ access to overdose response resources in BC.

Both our quantitative and qualitative results reveal the importance of having access to a cellphone for *GSDOA* awareness. Survey participants without cellphones had significantly lower odds of being aware of the *GSDOA*, and interviewees spoke of the difficulties of reaching PWUD who do not own a cellphone. Social media was recommended by interviewees to increase awareness of the *Act*, however, some participants were concerned that information posted online would be inaccessible to those without cellphones or access to the internet. This concern is supported by our quantitative findings, which indicated that 30% of participants did not have a cellphone and ownership was lower in adults (58%) than in youth (83%) (data not shown). Unsurprisingly, cellphone possession has been identified as an important factor in calling 9–1-1 in the event of an overdose [10, 15, 69]. In a sample of individuals released from correctional facilities, McLeod et al. [25] found unanimous willingness to call 9–1-1 for an overdose among those who had a cellphone. This highlights the value of increasing access to cellphones for people at risk of witnessing an overdose to promote help-seeking and access to educational materials pertaining to overdose response (e.g. information about the *GSDOA* available online).

Recent experiences with overdose also emerged as an important determinant of *GSDOA* awareness in our quantitative findings. Those who had witnessed an opioid overdose in the last 6 months were more likely to be aware of the *Act*. These individuals may be more likely to have been trained in overdose response and, as seen in our qualitative interviews, information about the *GSDOA* may have been included in such trainings. Additionally, these individuals may be more likely to be connected with overdose or treatment services that advertise the *Act* [70, 71]. Other studies have identified previously witnessing an overdose as a correlate of calling 9–1-1 [72]. Clearly, *GSDOA* awareness varies depending on the sub-population (e.g. age, recently in corrections, cellphone possession, recently witnessed opioid overdose) – pointing to the necessity of targeted knowledge translation interventions.

Since 2019, BC has aimed to increase *GSDOA* awareness and understanding by producing and disseminating knowledge translation materials. These included posters and videos at the federal level, posters and wallet cards at the provincial level, and brochures and factsheets locally by the non-profit organization PIVOT legal [17, 73–75]. Our qualitative findings indicated that some participants heard about the *GSDOA* through harm reduction and low-barrier service settings such as overdose prevention sites, shelters, healthcare services, and parole offices. This is supported by our quantitative finding that overdose response resources and characteristics were more important than demographic characteristics in explaining the variance in *GSDOA* awareness. This finding is in line with Mehta et al.’s study in BC which found greater odds of awareness among PWUD who frequently accessed harm reduction sites to obtain supplies [26], suggesting that some participants may have learned about the *GSDOA* through these sites. Although targeted knowledge translation at locations frequented by PWUD and people at risk of witnessing an overdose continues to be paramount, a variety of knowledge translation strategies are needed. Focused approaches may systematically exclude segments of the population such as those who are not accessing harm reduction informed or low-barrier services.

While some participants in our study reported learning about the *GSDOA* through materials, such as posters and videos, many participants shared that they had never seen *GSDOA* educational materials. To address this missed opportunity, other forums for information sharing, such as peer-to-peer contact-based education, should be explored. In addition, it may be beneficial to advertise *GSDOA* materials at a wider range of locations. For example, prior research in Vancouver suggests that posters distributed throughout a neighbourhood (e.g. telephone poles, bus stops) may be more impactful than posters confined to harm reduction sites and other services frequently visited by PWUD [76]. This is supported by our quantitative finding that awareness did not differ significantly in those who completed the survey online compared to in person. Standardized trainings may also be an appropriate platform for sharing information about the *GSDOA*. Our results found that the presence and amount of *GSDOA* information included in naloxone training sessions varied depending on the training and facilitator. Recently, information about the *GSDOA* was included in a standardized online training module for THN sites. More standardization is needed to ensure that information about the *Act* is routinely being disseminated as part of overdose response trainings and promotional materials.

*GSDOA* knowledge translation materials are available online on the Towards the Heart website. However, as both our quantitative and qualitative results demonstrated, a considerable proportion of people at risk of witnessing an overdose do not have access to cell phones with data or internet connectivity to use messaging services. Due to limited access to technology, qualitative interview participants expressed concerns about using social media and the internet to increase awareness and knowledge of the *Act*. Rather, word of mouth was highlighted as one of the most effective strategies to disseminate information widely among PWUD. Participants emphasized the element of trust present in information sharing through word of mouth delivered by peers that is missing from other mediums of knowledge translation. Our findings align with past research which shows that existing trusted relationships are a key element to effective knowledge dissemination among PWUD [77]. Lavis [78] highlights that a messenger who is credible and trusted is one of the most important aspects of knowledge translation. Existing research in BC shows that peers and people who deal drugs are often considered to be the most trustworthy sources of information about illicit drugs by PWUD – even when compared to roles that are traditionally associated with credibility, such as healthcare providers [76–78]. Future knowledge translation materials should be mindful of the messenger responsible for dissemination and consider leveraging existing, trusted interpersonal and organizational relationships.

Participants of all ages highlighted the need to include PWLLE in all knowledge translation efforts and expressed the importance of tailoring strategies to reach different audiences. Full engagement of PWLLE in every part of research and knowledge translation results in a process that is more equitable and effective [79]. Youth participants advocated for youth-appropriate knowledge translation approaches, such as educational sessions about the *Act* in schools, as well as awareness campaigns on social media. Although stigma and abstinence-based doctrines may pose a barrier to adding harm reduction informed material to school curricula, youth stressed the importance of challenging the stigma and informing all young people about the *GSDOA*, not just those considered as at-risk (e.g. street entrenched youth or youth who use drugs). Future research should focus on identifying and implementing youth-centred interventions. While these quantitative and qualitative findings report on *GSDOA* awareness and knowledge, the question remains: are drug-related Good Samaritan Laws having the intended outcome and encouraging people who witness an overdose to call 9–1-1? Research on the effectiveness of drug-related Good Samaritan Laws is limited and the results are mixed, with only some studies providing evidence that drug-related Good Samaritan Laws contribute to reducing illicit drug toxicity deaths [20, 27, 80]. Previous studies have found that awareness and complete knowledge of Good Samaritan Laws is associated with an increased likelihood of calling 9–1-1 [24–26, 29, 30, 68]. Even so, *GSDOA* awareness and understanding alone fail to account for other contextual factors that may impact an individual’s willingness to call 9–1-1 and therefore should not be used as a proxy for *GSDOA* effectiveness. For example, research shows that one’s willingness to call 9–1-1 for an overdose may also depend on factors such as overdose setting (private vs. public) and previous experiences with EHS [24, 81, 82]. Lastly, there is no conclusive evidence about the effectiveness of Good Samaritan Laws, in part due to ongoing concerns around police presence in spite of these laws [34, 36, 82]. Police discretion and stigma can cause PWUD to be poorly treated by first responders at overdose events [34, 36]. Negative experiences with first responders, including police officers, can erode PWUD’s trust and make people less likely to call 9–1-1 in the event of a future overdose [34, 36]. The *GSDOA* may need to be expanded through broader legally recognized, or *de jure*, decriminalization. Importantly, the challenges associated with ensuring *GSDOA* awareness and understanding described in this study are relevant to broader decriminalization and should be considered. More specifically, policy tenets need to be clear and concise to promote awareness and understanding. Although beyond the scope of this paper, a discussion of the impacts of police discretion and inconsistent approaches at overdose events can be found in Xavier et al. [39] and an exploration of PWUD’s perceptions of the *GSDOA*’s limitations and their relevance to discussions around broader decriminalization can be found in Xavier et al. [83]. It is important that future research explore determinants of calling 9–1-1 in regions with Good Samaritan Laws in a way that accounts for variable awareness and understanding of the law as well as different participant characteristics, such as gender, housing status, and socioeconomic status. Intention to call 9–1-1 should also be explored based on peoples’ level of concern with police including determinants such as arrest history and one’s perception of police officers.

### Strengths and Limitations

This multi-methods study provides a comprehensive examination of *GSDOA* awareness and understanding among a sample of people at risk of witnessing an overdose. Our findings add to the literature as they highlight the perspectives, awareness and understanding of people who are impacted by the *GSDOA* and can benefit from the legal protections the *Act* provides.

With that being said, our study has several limitations. We recruited participants by convenience sampling which likely affects the generalizability of the results. Our sample may not be representative of all people at risk of experiencing or witnessing an overdose across BC, considering recruitment for the quantitative survey was based around THN sites which may disproportionately represent people who access harm reduction services. Furthermore, the qualitative interviews required a time investment that may have deterred some potential participants. Self-selection bias may have been a contributing factor as participants who were willing to dedicate the time required to complete the qualitative interview may have been motivated by prior awareness of and interest in drug policy and the *GSDOA*.

Although attention was given to recruiting participants from diverse communities across BC, the majority were from relatively metropolitan, large urban, and medium urban areas. As such, findings may not be representative of people from rural jurisdictions in BC.

Due to COVID-19 public health and physical distancing guidelines, interviews were conducted over the phone. While PRAs provided participants with the option to borrow a phone or use their own, this process may have conceivably discouraged prospective participants as a level of pre-emptive planning was required to schedule the interview. Additionally, the rapport that can be established over the phone is limited, although research suggests that there is little difference in the quality of the data collected when compared to face-to-face interviews [84]. Though the *GSDOA* is a federal law, this study was conducted in BC and may not be applicable to other contexts, including other Canadian provinces as well as various states in the US that have enacted drug-related Good Samaritan Law. Finally, the majority of our measures were self-reported, which could introduce bias, namely social desirability bias, into our results.

## Conclusions

This study highlights that despite multiple knowledge dissemination efforts in BC, awareness and complete understanding of the *GSDOA* among people at risk of witnessing an overdose remains low. Misunderstandings of the *Act*’s protections can sow distrust in the *Act*, law enforcement, and harm reduction informed policy as a whole. Future efforts should involve targeted outreach to subgroups with low awareness and incomplete understanding of the *GSDOA* such as those who haven’t witnessed an opioid overdose and people who do not have access to a cellphone. Effective outreach strategies need to be led by people with lived and living experience of drug use and must incorporate peer-led strategies such as word of mouth to spread information. Additional research should explore the impact of Good Samaritan Laws on attitudes, intents, and behaviour around calling 9–1-1 at the scene of an overdose and determine the role of awareness and complete understanding.

## Supplementary Information

**Additional File 1.** Survey and Interview Materials

**Additional File 2.** Supplemental Figure and Table   

## Data Availability

The datasets analyzed during the current study are available from the corresponding author on reasonable request.

## Abbreviations

AIC: Akaike’s Information Criterion
AOR: Adjusted odds ratio
BC: British Columbia
BCCDC: British Columbia Centre for Disease Control
BCTHN: British Columbia Take Home Naloxone
CI: Confidence interval
EHS: Emergency health services
GSDOA: Good Samaritan Drug Overdose Act
MICE: Multiple imputation by chained equation
OR: Odds ratio
PRA: Peer Research Assistant
PWLLE: People with lived and living experience of substance use
PWUD: People who use drugs
US: United States
