# Supplementary material for: Awareness and knowledge of the Good Samaritan Drug Overdose Act among people at risk of witnessing an overdose in British Columbia, Canada: a multi-methods cross sectional study
Source: Subst Abuse Treat Prev Policy. 2022 May 25;17:42. doi: 10.1186/s13011-022-00472-4 (PMC9131579; doi:10.1186/s13011-022-00472-4)
Supplement: Supplementary file 2 — Additional File 2. Supplemental Figure and Table [file 13011_2022_472_MOESM2_ESM.docx]

**Additional File 2**

**
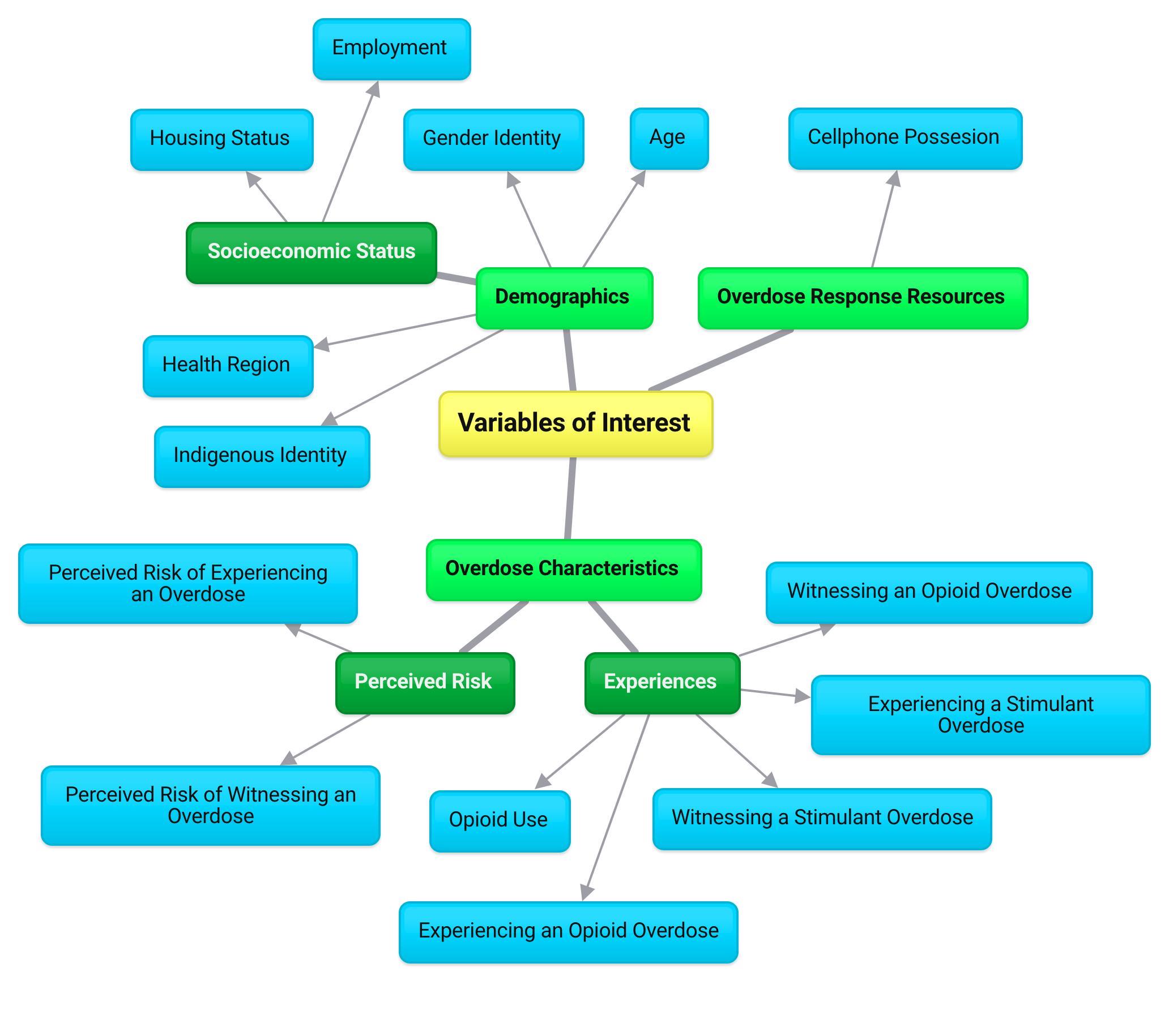
**

**Supplementary Figure 1.** Concept map of study variables of interest used to inform the development of a hierarchical regression model. Variables (blue) were assembled into categories (green) and subcategories (dark green) with categories ultimately becoming the “blocks” upon which the regression was built.

**Supplementary Table 1.** Factors associated with *GSDOA* awareness among survey respondents with “unknown” observations imputed by multiple imputation by chained equations (MICE).

|  | **GSDOA Awareness** | | |  |
| --- | --- | --- | --- | --- |
|  | **Aware**  (N = 259)  *n* (row %) | **Unaware**  (N = 234)  *n* (row %) | **Total**  (N = 493)  *n* (column %) | **P-value** |
| **Age (years)** |  |  |  | 0.138 |
| 16 – 24 years | 50 (43.9) | 64 (56.1) | 114 (23.1) |  |
| 25 – 34 years | 59 (61.5) | 37 (38.5) | 96 (19.5) |  |
| 35 – 44 years | 61 (55.5) | 49 (44.5) | 110 (22.3) |  |
| 45 – 54 years | 52 (51.5) | 49 (48.5) | 101 (22.3) |  |
| 55 years and over | 37 (51.4) | 35 (48.6) | 72 (14.6) |  |
| **Gender Identity** |  |  |  | 0.333 |
| Cis man | 141 (51.9) | 137 (48.1) | 285 (57.8) |  |
| Cis woman | 102 (55.1) | 83 (44.9) | 185 (37.5) |  |
| Trans and gender expansive | 9 (39.1) | 14 (60.9) | 23 (4.7) |  |
| **Indigenous Self-Identification** |  |  |  | **0.024** |
| Indigenous | 98 (46.4) | 113 (53.6) | 211 (42.8) |  |
| Non-Indigenous | 161 (57.1) | 121 (42.9) | 282 (57.2) |  |
| **Health Region** |  |  |  | 0.394 |
| Fraser | 54 (51.9) | 50 (48.1) | 104 (21.1) |  |
| Interior | 75 (58.6) | 53 (41.4) | 128 (26.0) |  |
| Island | 47 (47.0) | 53 (53.0) | 100 (20.3) |  |
| Northern | 21 (45.7) | 25 (54.3) | 46 (9.3) |  |
| Vancouver Coastal | 62 (53.9) | 53 (46.1) | 115 (23.3) |  |
| **Housing Status** |  |  |  | 0.924 |
| Private | 103 (52.3) | 94 (47.7) | 197 (40.0) |  |
| Supportive or Unstable Housing | 120 (53.3) | 105 (46.7) | 225 (45.6) |  |
| Homeless | 36 (50.7) | 35 (49.3) | 71 (14.4) |  |
| **Employment** |  |  |  | 0.751 |
| Yes | 84 (51.2) | 80 (48.8) | 164 (33.3) |  |
| No | 175 (53.2) | 154 (46.8) | 329 (66.7) |  |
| **Cellphone Possession** |  |  |  | **0.020** |
| Yes | 188 (56.3) | 146 (43.7) | 334 (67.7) |  |
| No | 71 (44.7) | 88 (55.3) | 159 (32.3) |  |
| **Perceived Risk of Experiencing an Overdose** (last 6 months)^a^ |  |  |  | **<0.01** |
| Never | 107 (44.8) | 132 (55.2) | 239 (48.5) |  |
| Ever | 152 (59.8) | 102 (40.2) | 254 (51.5) |  |
| **Perceived Risk of Witnessing an Overdose** (last 6 months)^a^ |  |  |  | **<0.01** |
| Never | 12 (20.7) | 46 (79.3) | 58 (11.8) |  |
| Ever | 247 (56.8) | 188 (43.2) | 435 (88.2) |  |
| **Opioid Use** (last 6 months) |  |  |  | **<0.01** |
| Yes | 175 (57.4) | 130 (42.6) | 305 (61.9) |  |
| No | 84 (44.7) | 104 (55.3) | 188 (38.1) |  |
| **Opioid Overdose** (last 6 months)^b^ |  |  |  | 0.153 |
| Yes | 60 (63.8) | 34 (36.2) | 94 (19.1) |  |
| No | 115 (54.5) | 96 (45.5) | 211 (42.8) |  |
| Didn’t use opioids | 84 (44.7) | 104 (55.3) | 188 (38.1) |  |
| **Stimulant Overdose** (last 6 months) |  |  |  | 0.189 |
| Yes | 49 (59.8) | 33 (40.2) | 82 (16.6) |  |
| No | 210 (51.1) | 201 (48.9) | 411 (83.4) |  |
| **Opioid Overdose Witnessed** (last 6 months) |  |  |  | **<0.01** |
| Yes | 190 (61.7) | 118 (38.3) | 308 (62.5) |  |
| No | 69 (37.3) | 116 (62.7) | 185 (37.5) |  |
| **Stimulant Overdose Witnessed** (last 6 months) |  |  |  | **<0.01** |
| Yes | 128 (61.2) | 81 (38.8) | 209 (42.4) |  |
| No | 131 (46.1)) | 153 (53.9) | 284 (57.6) |  |

^a^ “Never” = “Never”; “Ever” = “Rarely/sometimes/often/all the time”

^b^ “Didn’t use opioids” is shown but is not included in the chi square test

**Supplementary Table 2.** Adjusted odds ratios (AOR) for predictors of *GSDOA* awareness with multiple imputations by chained equation (MICE).

|  | **GSDOA Awareness** | |
| --- | --- | --- |
|  | Block 3 (OD Characteristics)^a^  AOR (95% CI) | Imputed Block 3 (OD Characteristics)^b^  AOR (95% CI) |
| **Demographic Characteristics** |  |  |
| Age (years) |  |  |
| 16 – 24 | — | — |
| 25 – 34 | 2.18 (1.09, 4.35) * | 1.92 (1.02, 3.64) * |
| 35 – 44 | 1.59 (0.81, 3.14) | 1.41 (0.77, 2.56) |
| 45 – 54 | 1.21 (0.62, 2.34) | 1.14 (0.62, 2.09) |
| 55 + | 1.37 (0.63, 2.95) | 1.41 (0.71, 2.81) |
| Gender |  |  |
| Cis man | — | — |
| Cis woman | 1.01 (0.63, 1.60) | 1.14 (0.75, 1.75) |
| Trans and gender expansive | 0.54 (0.17, 1.72) | 0.74 (0.28, 1.97) |
| **Overdose Response Resources** |  |  |
| Cellphone possession |  |  |
| Yes | 2.36 (1.44, 3.86) *** | 2.04 (1.29, 3.23) ** |
| No | — | — |
| **Overdose Characteristics** |  |  |
| Perceived risk of overdose^c^ |  |  |
| Ever | 1.47 (0.93, 2.31) | 1.48 (0.99, 2.21) |
| Never | — | — |
| Opioid overdose witness |  |  |
| Yes | 2.29 (1.42, 3.70) *** | 2.25 (1.46, 3.47) *** |
| No | — | — |

^a^Final unimputed model is based on N = 340 observations after exclusion of “unknown” responses for each variable.
^b^Final imputed model is based on N = 493 observations after “unknown” responses were imputed via MICE (Azur et al., 2011).
^c^“Never” = “Never”; “Ever” = “Rarely/sometimes/often/all the time”
